# A semi-analytical solution and AI-based reconstruction algorithms for magnetic particle tracking

**DOI:** 10.1371/journal.pone.0254051

**Published:** 2021-07-09

**Authors:** Huixuan Wu, Pan Du, Rohan Kokate, Jian-Xun Wang

**Affiliations:** 1 Department of Aerospace Engineering, School of Engineering, University of Kansas, Lawrence, Kansas, United States of America; 2 Department of Aerospace and Mechanical Engineering, University of Notre Dame, Notre Dame, Indiana, United States of America; Fuzhou University, CHINA

## Abstract

Magnetic particle tracking is a recently developed technology that can measure the translation and rotation of a particle in an opaque environment like a turbidity flow and fluidized-bed flow. The trajectory reconstruction usually relies on numerical optimization or filtering, which involve artificial parameters or thresholds. Existing analytical reconstruction algorithms have certain limitations and usually depend on the gradient of the magnetic field, which is not easy to measure accurately in many applications. This paper discusses a new semi-analytical solution and the related reconstruction algorithm. The new method can be used for an arbitrary sensor arrangement. To reduce the measurement uncertainty in practical applications, deep neural network (DNN)-based models are developed to denoise the reconstructed trajectory. Compared to traditional approaches such as wavelet-based filtering, the DNN-based denoisers are more accurate in the position reconstruction. However, they often over-smooth the velocity signal, and a hybrid method that combines the wavelet and DNN model provides a more accurate velocity reconstruction. All the DNN-based and wavelet methods perform well in the orientation reconstruction.

## 1. Introduction

Optical-based particle tracking technologies provide crucial knowledge and experimental guidance in the study of turbulence and complex flows [1, 2]. However, the advanced optical approaches face severe problems and cannot be used in many opaque environments such as granular motion and dense particulate flows. Missing the experimental guidance, to some extent, retards the development of granular dynamics and multiphase flow theory. A group of non-optical tracking methods is therefore developed, e.g., radioactive particle tracking (RPT) and positron emission particle tracking (PEPT) [3–10], but they encounter new issues. For instance, they require expensive equipment and special expertise for radioactive material operation. The magnetic resonant imaging (MRI) has a limited temporal resolution [11], which is insufficient for high-speed motion measurements.

A new magnetic particle tracking (MPT) technology has recently been developed to address the issues facing the non-optical methods [12–20]. The working principle of MPT is to locate a magnetic source according to its field, which is modeled as a dipole. The MPT has the following advantages: 1) it is a safe technique involving no radiation; 2) it provides not only the translation but also the rotation information of a particle, which is critical to granular dynamics; 3) it has a sufficient temporal resolution to measure high-speed flow; 4) it is cost-efficient as the magnetometers and sensors are much less expensive than the equipment in other non-optical approaches.

The key to the MPT method lies in the reconstruction of the position and orientation of a magnetic dipole. In other words, given the measurements (***B***_***1***_, ***B***_***2***_, …) at a few points (or field gradient ∇***B***), what is the position ***x*** and moment ***m*** of the magnetic source? The dipole field equation is $B=\frac{\mu_{0}}{4\pi }\frac{\left[3n∙(m∙n)-m\right]}{|x{|}^{3}}$, where ***n*** is the unit vector in the ***x*** direction. In experimental applications, numerical optimization and filtering are widely used to calculate ***x*** and ***m***, but these methods can be time-consuming and usually rely on artificial parameters or thresholds [12, 18, 20, 21]. In contrast, if the dipole field equation can be inverted, we can find a set of analytical solutions ***x*** = ***x***(***B***_***1***_, ***B***_***2***_,…) and ***m*** = ***m***(***B***_***1***_, ***B***_***2***_,…), which provides an efficient way to directly calculate ***x*** and ***m*** without any parameters. In the early stage, a group of researchers developed the eigenvector method [22] and Nara method [23]. Later on, a scalar triangulation and ranging (STAR) method was proposed for real-time magnetic target localization [24], and this method was modified multiple times [25]. These analytical methods possess a clear physical meaning and have been used in practical problems. However, they involve the field gradient tensor ∇***B***, which casts special requirements on the magnetometer and the sensor arrangement. In addition, the STAR method has a larger asphericity error [26, 27], and its modifications may involve a complex sensor setup [28].

In order to provide an accurate reconstruction method, this paper describes a new analytical solution that can be used for an arbitrarily arranged 3-axis magnetometer array. The reconstruction algorithm is accurate because it contains no assumptions other than the dipole model. However, for reconstruction in practical applications, denoising is an indispensable step since a real measurement contains uncertainty. The classic trajectory denoising method uses linear filtering [19, 29]. Given the measured location *y*, the filtered position is *x*(*t*) = ∫ *y*(*t* − *τ*)*K*(*τ*)*dτ*, where *K* is an integration kernel (e.g., a Gaussian kernel). Although this method is simple, the kernel used is fixed and cannot adapt to any temporal (or spatial) variation. To take care of the variation, more sophisticated denoising algorithms based on time-frequency decomposition have been developed. The general process of these approaches is twofold: (1) the original signal is transformed into the frequency domain, where the clean signal and noise can be represented by sparsely distributed spectral coefficients; (2) then a threshold scheme is applied to trim off noisy components, and the remaining are transferred back into the time domain for signal reconstruction. Wavelet transform (WT) is one classic example of time-frequency based methods and has been applied to a wide array of image denoising problems due to its advantage in removing random noise and improving the signal-to-noise ratio (SNR), even when noise and signal frequencies overlap [30]. Unfortunately, an appropriate threshold scheme is often hard to determine in the presence of residual or drifting noise [31]. Recent attempts for denoising problems focus on AI-based algorithms, which are capable of suppressing drifting noise and capturing local features robustly given labeled training data. Vincent et al. [32] constructed a denoising auto-encoder (DAE) neural network, aiming to find robust representations of features from noisy input data. Subsequent works are dedicated in optimizing deep neural network (DNN) structures to achieve better performance in handling complex noise and interference [33–36]. Representative network structures include fully-connected multilayer perceptron (MLP), convolutional neural networks (CNN), and recurrent neural networks (RNN) such as long short-term memory (LSTM) net. The CNN-based methods are typically implemented in an encoding-decoding fashion, where latent features are first extracted by the encoder layers and details are then compensated by the decoder layers to recover a clean version of the original signal [37]. Another popular trend is to utilize RNN to preserve historical information and temporal coherence while denoising, which is effective when handling sequential data, e.g., time series [38]. In this work, we design a novel denoising algorithm by leveraging both unsupervised WT and supervised RNN models with gated recurrent units (GRU), aiming to reduce the noise of the particle trajectory and orientation time series. Using synthetic data, we evaluated the reconstruction performance by comparing it with pure WT, CNN and GRU denoising methods.

The rest of the paper is organized as follows. We first describe the proposed 2D analytical solution and generalize it to 3D in Section 2. Using synthetic data, we evaluate the reconstruction accuracy in Section 3 and then investigate the performance of the proposed denoising scheme in Section 4, where artificial noises are considered. Finally, Section 5 concludes the paper.

## 2. Analytical solution

### 2.1 The 2D solution

**Fig 1:**
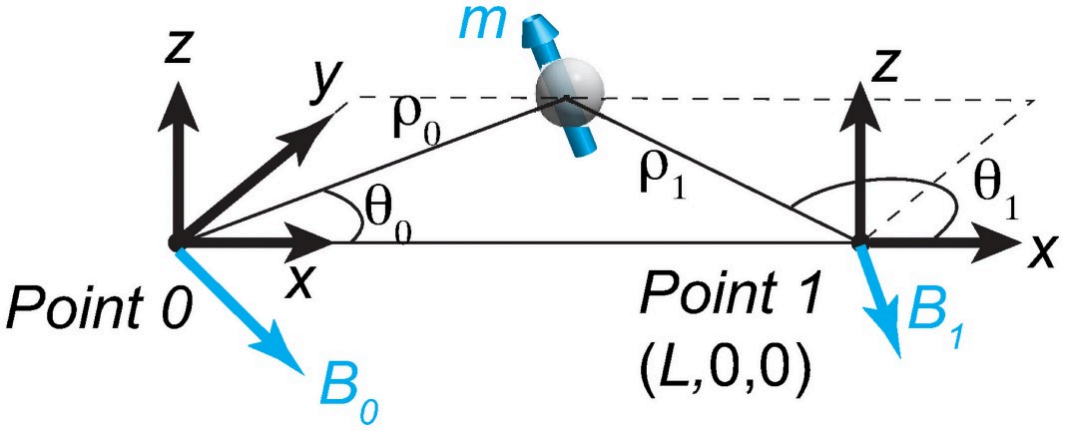
The magnetometer arrangement.

In this section, we first discuss the analytical solution to a restricted problem. In the simple setup shown in Fig 1, there are two 3-axis magnetometers located at Point 0 and 1 with ***x***_*0*_ = (0, 0, 0) and ***x***_1_ = (*L*, 0, 0). They together provide six signals (*B*_0x_, *B*_0y_, *B*_0z_) and (*B*_1x_, *B*_1y_, *B*_1z_). The magnetic particle moves on the *x-y* plane (z = 0), but its magnetic moment is 3D, ***m*** = (*m*_*x*_, *m*_*y*_, *m*_*z*_)′. According to the dipole field model, the magnetic field strengths at ***x***_*0*_ and ***x***_1_ are $B(x_{0,1})=\frac{\mu_{0}}{4\pi }\frac{\left[3n∙(m∙n)-m\right]}{|x-x_{0,\;1}{|}^{3}}$, where ***x*** is the magnet’s location, **n** is the normal vector in the ***x–x***_*i*_ direction (*i* = 0 or 1), ***m*** is the magnetic moment, and *μ*_0_ is the magnetic permeability. The dipole field is exact for a uniform spherical magnetic bead, and it is a very good approximation for the far field of non-spherical beads [39]. Since ***x–x***_i_ lies on the *x-y* plane and **n**_i_ = (*n*_*ix*_, *n*_*iy*_, 0)′, the magnetic field equation is

BixBiyBiz=μ04πx-xi33nixmxnix+myniy-mx3niymxnix+myniy-my-mz (1)

where the index *i* is 0 or 1.

To calculate ***m*** and ***x***, the first step is to eliminate the nonlinear term |***x*** − ***x***_***i***_|^3^. Generally, the *z* components (*B*_*z*_ and *m*_*z*_) are not zero. Thus, we can normalize the *x* and *y* equations using the *z* component and define **M** = (*M*_*x*_, *M*_*y*_)’ = (*m*_*x*_ */m*_*z*_, *m*_*y*_ */m*_*z*_)’ and **T**_i_ = (*T*_*ix*_, *T*_*iy*_)’ = (*B*_*ix*_/*B*_*iz*_, *B*_*iy*_/*B*_*iz*_)′. Hence, **T**_i_ = 3**n**_i_(**M** · **n**_i_) − **M**. The *B*_*z*_ = 0 case will be discussed later. Define a unit vector ***t***_***i***_ in the *x-y* plane, **t**_i_ = (−*n*_*iy*_, *n*_*ix*_, 0)′, so **t**_***i***_ is normal to **n**_**i**_, i.e., **t**_**i**_ · **n**_**i**_ = 0. Now we decompose the vector **T**_i_ to the **t**_**i**_ and **n**_**i**_ directions:

$$T_{i}\cdot n_{i}=3\left(n_{i}\cdot n_{i}\right)\left(M\cdot n_{i}\right)-M\cdot n_{i}=2M\cdot n_{i}$$ (2.1)

as ***n*** is a unit vector, and

$$T_{i}\cdot t_{i}=-M\cdot t_{i}$$ (2.2)

Substitute the components of each vector, we obtain

$$\left(\begin{array}{cc} T_{ix}-2M_{x} & T_{iy}-2M_{y}\\ T_{iy}+M_{y} & -T_{ix}-M_{x} \end{array}\right)\cdot \left(\begin{array}{c} n_{ix}\\ n_{iy} \end{array}\right)=0$$

If this equation has a non-zero solution, the coefficient matrix must have a zero determinant. Hence,

$$\left(T_{ix}-2M_{x}\right)\left(T_{ix}+M_{x}\right)+\left(T_{iy}-2M_{y}\right)\left(T_{iy}+M_{y}\right)=0$$

After rearranging, we get

$${\left({\frac{1}{4}T}_{ix}+M_{ix}\right)}^{2}+{\left(\frac{1}{4}T_{iy}+M_{iy}\right)}^{2}=\frac{9}{16}\left(T_{ix}^{2}+T_{iy}^{2}\right)$$ (3)

**Fig 2:**
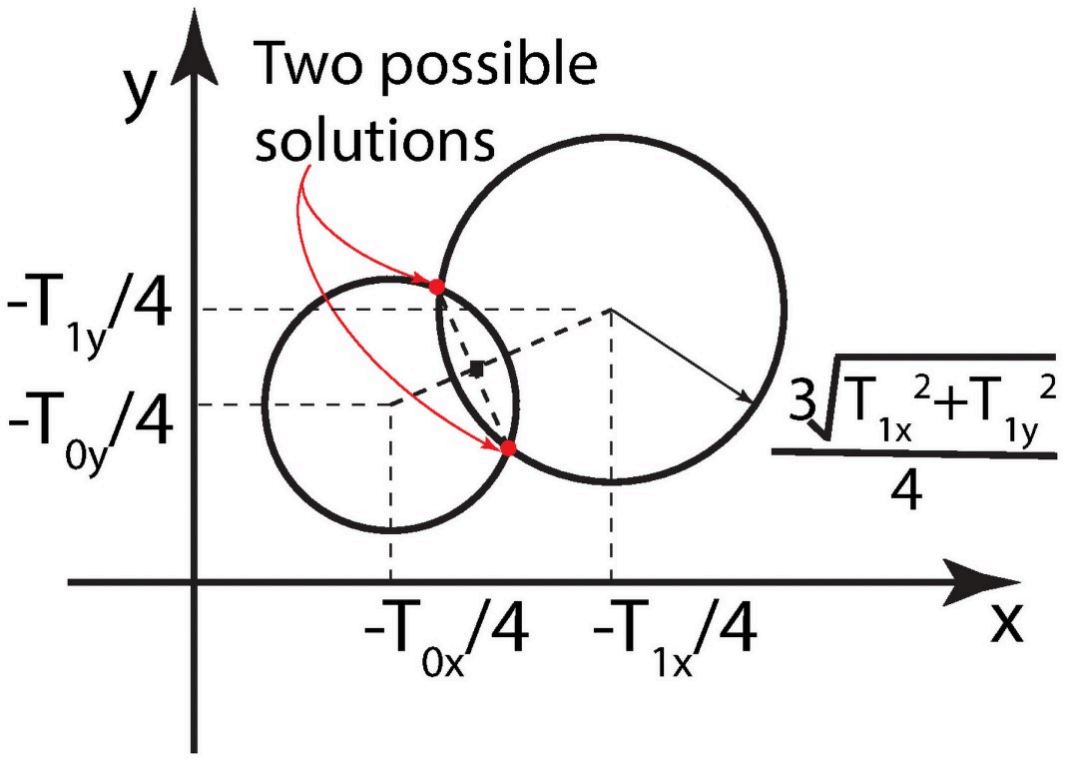
Two circles described by Eq 3. The joints are candidate solutions to **M**.

Note that the values of **T** components are known and fixed in a measurement, and **M** is unknown. Eq 3 describes two circles for *i* = 0 and 1. The radius is $\frac{3}{4}{\left(T_{ix}^{2}+T_{iy}^{2}\right)}^{1/2}$ and the center is at (–*T*_*ix*_/4, –*T*_*iy*_/4), as illustrated in Fig 2.

These two circles have two joints, one of which is the solution **M**. Based on the geometric relationship, we can get two candidate solutions:

M=mxmzmymz=Mx*My*±ST0x-T1x2+T0y-T1y2T0y-T1y-T0x+T1x (4)

where $S=\frac{1}{2}{\left[T_{0x}^{2}+T_{1x}^{2}+T_{0y}^{2}+T_{1y}^{2}+\left(T_{0x}+T_{1x}\right)M_{x}^{*}-4M_{x}^{*2}+\left(T_{0y}+T_{1y}\right)M_{y}^{*}-4{M_{y}^{*}}^{2}\right]}^{1/2},\;M_{x}^{*}=\frac{\left(T_{0x}-T_{1x}\right)D-\left(T_{0y}-T_{1y}\right)E}{{\left(T_{0x}-T_{1x}\right)}^{2}{+\left(T_{0y}-T_{1y}\right)\;}^{2}}$ and $M_{y}^{*}=\frac{\left(T_{0y}-T_{1y}\right)D+\left(T_{0x}-T_{1x}\right)E}{{\left(T_{0x}-T_{1x}\right)}^{2}{+\left(T_{0y}-T_{1y}\right)\;}^{2}}$. Here, $D=T_{1y}^{2}-T_{0y}^{2}+T_{1x}^{2}-T_{0x}^{2}$ and E = (*T*_0x_*T*_1y_ − *T*_1x_*T*_0y_)/4.

Finally, we need to choose the correct solution from the two possible results. To proceed, selecting one solution **M**, we can obtain tan *θ*_0_ = (*T*_0*y*_ − *M*_*y*_)/(*T*_0*x*_ − *M*_*x*_) and tan *θ*_1_ = (*T*_1*y*_ − *M*_*y*_)/(*T*_1*x*_ − *M*_*x*_). Here *θ*_*i*_ is the angle between **n**_**i**_ and the *x*-axis (Fig 1). Consequently, the magnet’s position is

$$x=L\;\text{tan}\;\theta_{1}/(\;\text{tan}\;\theta_{1}-\;\text{tan}\;\theta_{0})$$ (5.1)

$$y=\;\text{tan}\;\theta_{0}∙x$$ (5.2)

Hence, the position ***x*** = (*x*, *y*, 0)’, and the magnitude |***x*** − ***x***_*i*_|^3^ can be determined. Thereafter, the *z*-component equations *B*_0*z*_ = −*μ*_0_*m*_*z*_/4*π*|***x*** − ***x***_0_|^3^ and *B*_1*z*_ = −*μ*_0_*m*_*z*_/4*π*|***x*** − ***x***_1_|^3^ provide two possible *m*_*z*_’s with corresponding (*m*_*x*_, *m*_*y*_)’ = *m*_*z*_·**M**. If the selected **M** is wrong, the two *m*_*z*_’s calculated using *B*_0z_ and *B*_1z_ do not match and this solution should be discarded. In addition, if the magnitude of ***m*** is known, we can also determine the correct solution by comparing the magnitude of reconstructed ***m*** with the known value.

Now, we discuss the *m*_*z*_ = 0 case. The above reconstruction algorithm can be written briefly as functions ***x*** = ***x***(**B**_0_, **B**_1_) and **m** = **m**(**B**_0_, **B**_1_). They are finite for any *m*_*z*_ = ± ε, where 0 < ε << 1. As continuous functions that describe a physical trajectory, the left and right limits must be equal, ${\text{lim}}_{B_{0z}\to 0^{-},\;B_{1z}\to 0^{-}}x\left(B_{0},B_{1}\right)={\text{lim}}_{B_{0z}\to 0^{+},\;B_{1z}\to 0^{+}}x(B_{0},B_{1})$. In other words, the B_0z_ = B_1z_ = 0 case is a removable singularity of the function. In real applications, the probability of measuring an exact zero *m*_*z*_ is practically zero, so the above solution works for nearly all measurements.

### 2.2 From 2D to 3D

**Fig 3:**
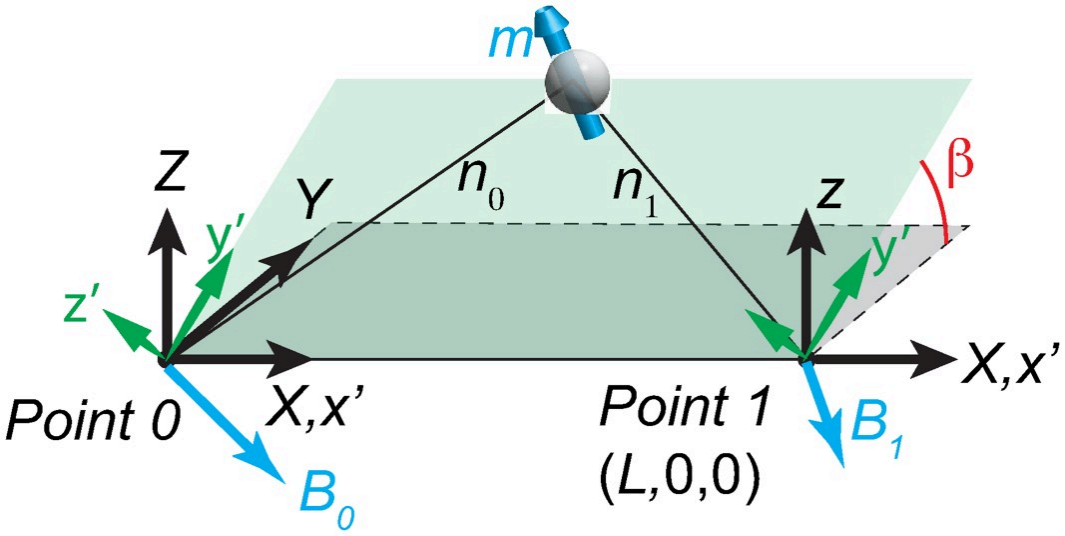
The 3D setup and coordinate definition.

The above 2D analytical solution can be generalized to 3D with an arbitrary sensor arrangement. As shown in Fig 3, the magnet is located on the *x’-y’* plane, which has a *β* angle from the *X-Y* plane in the 3D space. The *x’* and *X* axes overlap. The angle *β* is regarded as a parameter to be determined later. If we rotate the frame of reference from *XYZ* to *Xy’z’*, the 3D reconstruction reduces to the 2D problem. After the rotation, the magnetometer readings become

$$B^{\prime }=B^{\prime }\left(\beta \right)=R\left(\beta \right)\cdot B=\left(\begin{array}{ccc} 1 & 0 & 0\\ 0 & \text{cos}\;\beta & -\text{sin}\;\beta \\ 0 & \text{sin}\;\beta & \text{cos}\;\beta \end{array}\right)\cdot \left(\begin{array}{c} B_{X}\\ B_{Y}\\ B_{Z} \end{array}\right)$$ (5)

Substituting **B’** into the 2D solutions (Eqs 3 and 4), we can reconstruct the status of the magnet with the parameter *β*: ***x*’** = ***x*’(B’) = f**(**B**, *β*) and **m’** = **m’(B’) = g**(**B**, *β*). Then, the position and moment in the original *XYZ* frame is

$$x=R^{-1}\cdot x^{\prime }=R^{-1}\cdot f(B,\beta )$$ (6.1)

$$m=R^{-1}\cdot m^{\prime }=R^{-1}\cdot g(B,\beta )$$ (6.2)

Finally, we need to determine the parameter *β* by substituting ***x*** and **m** to the dipole equation. Define $h\left(B,\beta \right)=\frac{\mu_{0}}{4\pi }\frac{\left[3n∙\left(m∙n\right)-m\right]}{|x-x_{0,\;1}{|}^{3}}$, where ***x*** and ***m*** are functions of **B** and the unknown parameter *β*, the dipole equation becomes

$$0=||B-h\left(B,\beta \right)|{|}_{2}$$ (7)

**Fig 4:**
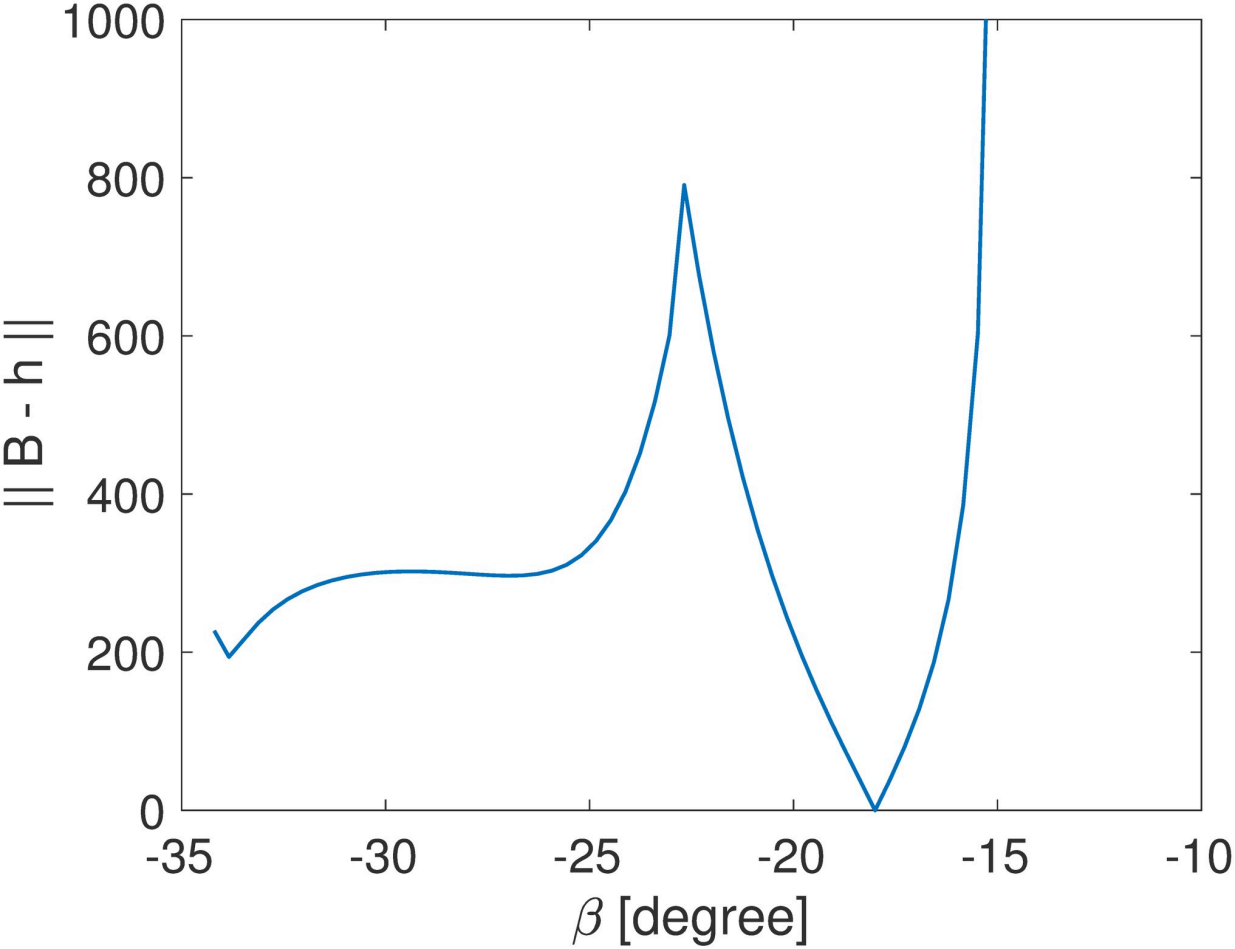
A sample curve illustrates the relationship between ||B–h|| and *β*.

On the right hand side, the field strength **B** is known. Recall that the 2D solution contains two possible results, so the ||**B–h**|| curves may include two branches, but only one of them reaches zero. Fig 4 shows a sample curve of ||**B–h**|| as a function of *β*.

The function **h** is highly non-linear, but it contains only one variable, as **B** are known. Therefore, it is actually efficient to solve Eq 7 using Newton’s method in a practical problem, and the reconstructed position in a previous step (a known *β*) can be used as the initial estimation for the next time step.

For a 3D problem with an arbitrary 3-axis-sensor arrangement (each magnetometer should measure all three components of **B**), we can apply the above method to any pair of sensors and calculate the averaged reconstruction. In other words, if there are *N* magnetometers, they form N2 pairs. Each pair produces an ***x*** and ***m***. The final reconstruction can be obtained by averaging ***x*** and ***m***, which reduces errors in the results. Ideally, the uncertainty decreases as $\frac{1}{\sqrt{N\left(N-1\right)/2}}$ at a large N. This is better than the methods in literature, where the uncertainties scales as $\frac{1}{\sqrt{N}}$. However, the reconstruction speed of our method is lower.

## 3. Trajectory reconstruction

### 3.1 Trajectory generation and reconstruction

**Fig 5:**
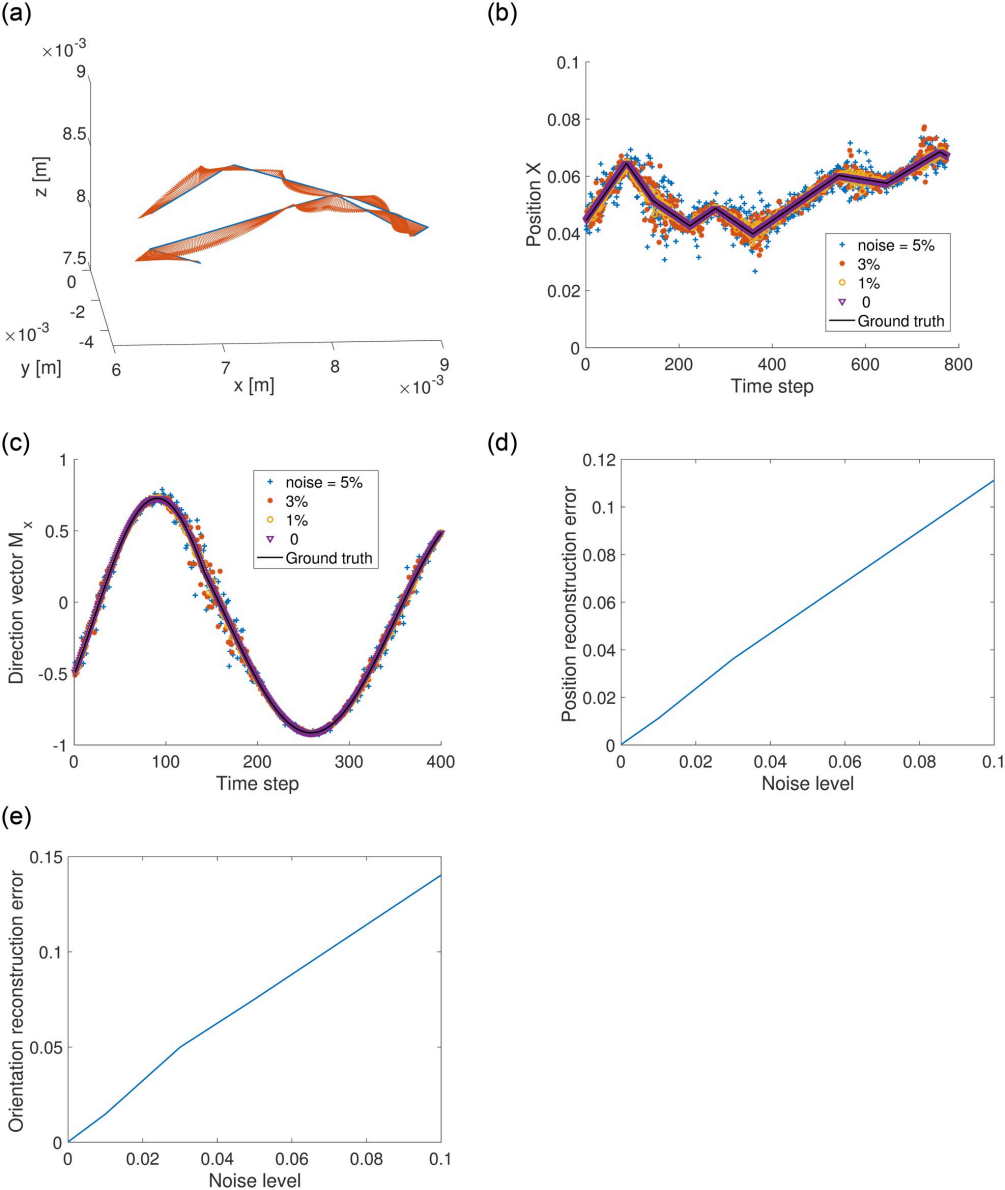
a) A sample synthetic trajectory that contains sudden velocity changes and turns. The arrows indicate the magnetic moment direction. b) The original and reconstructed positions at various noise levels. The unit of *x* is meter. c) The ground truth and reconstructed orientations. Here the orientation is normalized using the magnitude |**m**|. d) The position errors at various noise levels. The error is normalized using the measurement domain size *L*. e) The orientation errors at different noise levels. The error is normalized using |**m**|.

In this section, we will investigate the reconstruction accuracy using synthetic trajectories. The MPT method can be used to study, e.g., fluidization, where collisions among particles can be intense and hard to measure. Therefore, we design the trajectories to contain random turns and sudden velocity changes. Fig 5a shows one sample trajectory section in a 3D space. The domain is a cube with the boundary size L = 0.1 m. One magnetometer is located as the origin and the other is at (L, 0, 0). The directions of the sensors are aligned with the frame of reference (*XYZ*). This setup has been plotted in Fig 3. Given the trajectory, we can simulate the magnetometer readings using the dipole model. Since real measurements always contain uncertainty, we model the field noise using a Gaussian distribution,

$$B_{x,y,or\;z}={\hat{B}}_{x,y,or\;z}(1+\varepsilon )$$

where $\hat{B}$ is the ground truth obtained from the synthetic trajectory, and *ε* is a random variable with zero-mean normal distribution. Here we investigate a series of noise levels, beginning with ε = 0 (no noise) to *std*(ε) = 0.01, 0.03, 0.05, 0.1, … 0.3, where *std* means the standard deviation.

Fig 5b and 5c show a sample of reconstructed position ***x*** and orientation ***m***_*x*_. At the zero noise level, our method in Section 2 can reconstruct the position and orientation of a magnet with no error. The zero-noise reconstructed trajectory overlaps the ground truth curve. As the measurement noise increases, the reconstruction error increases. For example, when *std*(ε) = 0.05, the reconstructed positions deviate from the ground truth significantly. We define the position error as the mean deviation normalized by the measurement domain size, $mean(\left|\hat{x}-x\right|)/L$. Fig 5d shows the position error’s dependence on the measurement noise, which is nearly a straight line. For a typical sensor noise level of 3%, the reconstruction error is roughly 3–4% of the domain size, which is consistent with the filtering method [20]. The orientation reconstruction error, defined as $mean(\left|\hat{m}-m\right|)/|m|$, is shown in Fig 5e.

### 3.2 Propagation of uncertainty

The reconstruction error is highly inhomogeneous in the spatial and orientation domain. This is caused by the propagation of error in the reconstruction. To provide a clear explanation, we differentiate Eq 1, $\delta B_{i}=\frac{\partial B_{i}}{\partial x_{j}}\delta x_{j}+\frac{\partial B_{i}}{\partial m_{k}}\delta m_{k}$. Written in a matrix format, we have *δ***B** = Pδ**V**. *δ***B** = (*δ*B_0x_, *δ*B_0y_, *δ*B_0z_, *δ*B_1x_, *δ*B_1y_, *δ*B_1z_)′ can be regarded as the measurement noise and δ**V** = (*δx*, *δy*, *δz*, *δm*_*x*_, *δm*_*y*_, *δm*_*z*_)′ is the reconstruction error, where prime means array transpose. The entry in the matrix **P** is $P_{ij}=\frac{\partial B_{i}}{\partial x_{j}}$ (for *j* ≤ 3) or $\frac{\partial B_{i}}{\partial m_{j}}$ (for *j* ≥ 4), which is a function of ***x*** and ***m*** reconstructed using the **B**. Define the invert of **P** as **W = P**^–1^, we obtain δ**V** = Wδ**B**. The position reconstruction error is therefore

$$\delta x=W_{1}\delta B$$

and the orientation error is

$$\delta m=W_{2}\delta B$$

where W_1_ is the first three rows of W, and W_2_ is the 4^th^-6^th^ rows. The maximum ||δ***x***|| can hence be estimated as,

$${\left|\left|\delta x\right|\right|}_{max}=\sqrt{\delta B^{\prime }\cdot W_{1}^{\prime }W_{1}\cdot \delta B}\;\sim \;\sigma_{1}|\left|\delta B\right||$$ (8.1)

where σ_1_ is the square root of the maximum eigenvalue of $W_{1}^{\prime }W_{1}$. A similar argument shows that the max ||δ**m**|| is

$${\left|\left|\delta m\right|\right|}_{max}\;\sim \;\sigma_{2}|\left|\delta B\right||$$ (8.2)

where σ_2_ is the square root of the maximum eigenvalue of $W_{2}^{\prime }W_{2}$. The coefficient σ_1_ and σ_2_ characterizes the propagation of error in the reconstruction.

**Fig 6:**
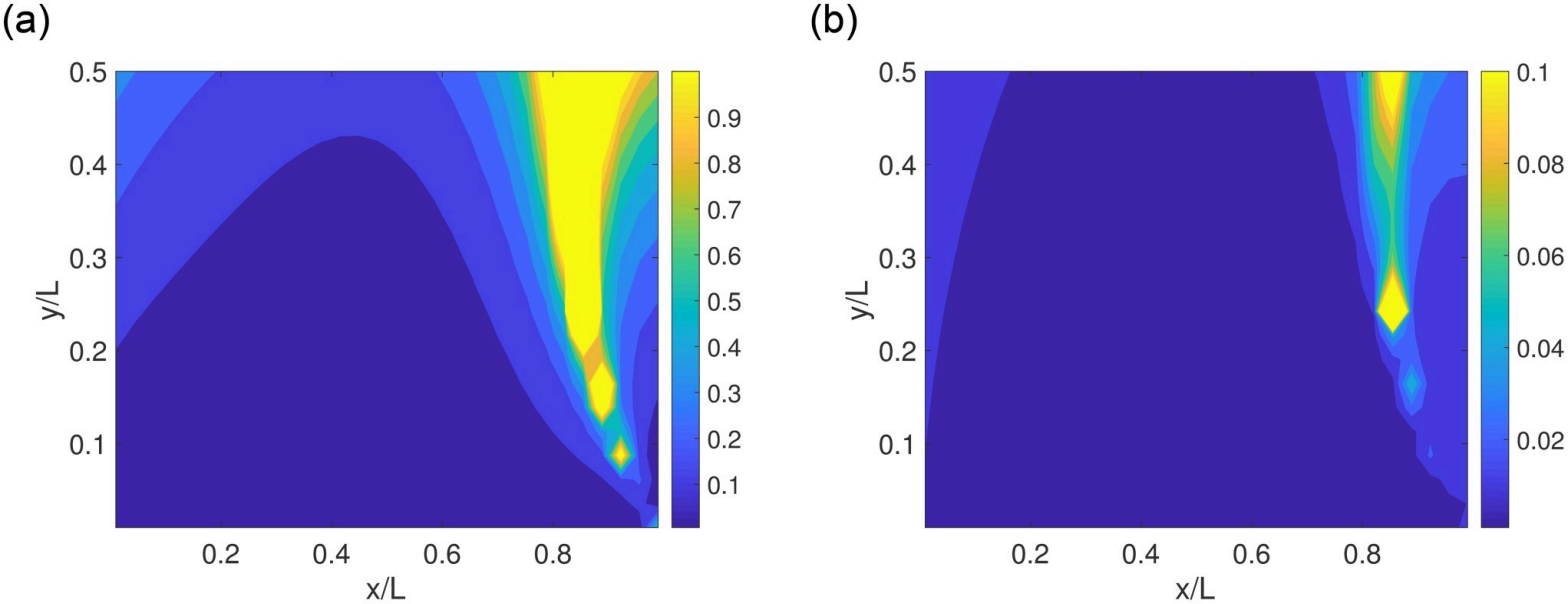
The distributions of a) σ_1_ and b) σ_2_, which characterize the reconstruction error in position and orientation, respectively. The unit of σ_1_ is [mm/μT] and that of σ_2_ is [Am^2^/μT]. Here L = 0.1 m, |**m**| = 0.001Am^2^ and pointing at the (1, 1, 1) direction.

To illustrate the spatial distribution of the error propagator, we select a specific case, in which the magnetic moment is |**m**| = 0.001Am^2^ and it points at the (1, 1, 1) direction. The noise level of **B** measurements in a L = 0.1 m domain is in the order of micro Tesla (μT). Fig 6 shows σ_1_ and σ_2_ on a sample plane (*z* = 0 plane). Note that we present σ_1_ and σ_2_ with their metric units because the **B** vary significantly and normalization with one factor can be misleading. The results suggest that there are a few regions with high σ_1_ and σ_2_ values. Physically, in these regions, the magnetic field strength **B** are insensitive to at least one component of ***x*** or **m**. As a result, the matrix **P** becomes nearly singular and the eigenvalues σ_1_ and σ_2_ become very large. Hence, if the particle trajectory passes through these regions, the uncertainty is larger, which can be manifested as a burst of reconstruction error. In real applications, this error can be removed using more magnetometers in an array.

## 4. Denoising using wavelet transform and deep neural network

To examine the performance of WT and AI-based denoisers, we utilize them to process 2000 Lagrangian trajectories that are reconstructed from synthetic signals with 3% random noise. The reconstruction follows the two-magnetometer setting illustrated in Fig 3.

### 4.1 AI- and WT-based denoising method

Two types of DNN structures (i.e., CNN and GRU) are studied for developing the AI-based denoiser. The first proposed denoising method is based on a CNN autoencoder, which contains two 1-D convolutional layers encoding the noisy signal into the latent space and two 1-D deconvolution layers decoding the latent signal to the denoised one after a linear layer. Each convolution/deconvolution layer has a kernel size of 3×1 and stride size of one, followed by a rectified linear unit (ReLU) activation function to account for nonlinearity (more details can be found in Table 1). The convolutional and deconvolutional layers have symmetric parameters such that the output has the same dimension as the input. The noisy trajectory data are fed into the CNN autoencoder with a size of 500×3. The denoised signals can be obtained by the forward evaluation of the network after sufficient training. The second AI-based model uses GRU to preserve the long-term memory of the sequence data. After conducting our preliminary studies on the synthetic data, the GRU is chosen over the LSTM because GRU achieves a close performance to LSTM with less trainable parameters, hence higher efficiency. The DNN within the denoiser starts with two GRU layers stacked together, which convert three channels into nine channels in the hidden layer, and ends with a linear layer that reduces the dimension back to three channels. The input data structure and implementation of the GRU-based model are identical to that in the aforementioned CNN-based model. More details about the GRU parameters can be found in Table 1. Both DNN-based denoisers are implemented in PyTorch, which is an open-source python platform for machine/deep learning.

**Table 1:** DNN structure.

DNN type	Layers	Characteristics
CNN	Convolution layer 1d	Kernel size = 3, stride = 1, input feature = 3, output feature = 12
	ReLU	-
	Convolution layer 1d	Kernel size = 3, stride = 1, input feature = 12, output feature = 48
	ReLU	-
	Deconvolution layer 1d	Kernel size = 3, stride = 1, input feature = 48, output feature = 12
	ReLU	-
	Deconvolution layer 1d	Kernel size = 3, stride = 1, input feature = 12, output feature = 9
	ReLU	-
	Linear layer	Input feature = 9, output feature = 3
GRU	GRU layer	Input feature = 3, output feature = 9
	GRU layer	Input feature = 9, output feature = 9
	Linear layer	Input feature = 9, output feature = 3

As mentioned, a synthetic dataset of 2000 trajectories is built for training and testing the DNN-based denoiser. The whole dataset contains 2000 samples, each of which has reconstructed results with artificial noise introduced by the synthetic sensor and the corresponding clean signal as ground truth. The datasets are divided into 1800 samples for training and 200 samples for testing. The training dataset is split into mini batches with a size of 200, which is randomly shuffled, for the neural network to update the model parameters in each epoch. Both CNN and GRU models are trained on an NVIDIA GTX2080Ti GPU using the Adam optimizer with a constant learning rate of 0.001. Each training needs at least 1200 epochs to converge, which takes roughly 0.5 hours on the single GPU. For example, the CNN can achieve a root mean square error (RMSE) of 0.0021 after 1200 epochs of training which takes about 0.58 hours.

In addition, a WT denoising method is also developed for comparison purpose, which is based on the discrete wavelet transformation (DWT) described as follow,

$$X\left(k\right)=\sum_{n=-\infty }^{+\infty }x(j)g(Cj-k)$$ (9)

where *X*(k) are the DWT coefficients, *x*(*j*) and *g*(*j*) represent the input signal and wavelet filters respectively, *k* is the shift coefficient, and *C* is the scaling factor (normally chosen as 2). The WT model uses VisuShrink [40], which applies a global threshold defined as $\gamma \sqrt{2logQ}$, where γ and *Q* denote the noise variance and number of signal elements (or image pixel), respectively. VisuShrink threshold is renowned for its capability of removing Gaussian noise with high probability and therefore widely applied in image denoising problems. Our WT model adopts Coiflet wavelet basis function with wavelet level set to five and uses soft threshold mode. The WT transformation is implemented by using scikit-image [41] in python and applied on the synthetic data regarding each time series as a 1D image. Note that, different from the AI-based denoisers, which require the clean signals as labeled data to learn from, the WT-based denoiser directly rejects noise in the given signal without any labels. However, as mentioned above, the WT-based approaches have difficulties dealing with residual noises, and it is hard to specify appropriate filter banks and suitable hyperparameters that work for all samples.

We define a metric to describe the relative noise strength, referred to as noise level (N),

$$N=\frac{\left|\left|X_{noisy}-X_{clean}\right|\right|}{\left|\left|X_{clean}\right|\right|}$$ (10)

where ***X***_*noisy*_ and ***X***_*clean*_ represent the noisy and clean signals, respectively. Considering that the noisy signal has large fluctuations of high frequency, a second metric is introduced to evaluate the extent of fluctuation in the following form,

$$N_{f}=\frac{\left|\left|{\dot{X}}_{noisy}-{\dot{X}}_{clean}\right|\right|}{\left|\left|{\dot{X}}_{clean}\right|\right|}$$ (11)

where $\dot{X}$ represents the forward finite difference of a time series ***X***. Note that if ***X*** represents the velocity, $\dot{X}$ is the acceleration. The performance of a denoising model can be characterized by the signal improvement ratio,

$$I=\frac{N_{noisy}-N_{denoised}}{N_{noisy}}\times 100\%$$ (12)

Due to significant noise level in velocity, the signal is plotted in the symmetric pseudo log-scale to resize the continuous real value space of velocity axis,

$$f\left(x\right)=sign\left(x\right)*\text{log}(1+x/10^{C})$$ (13)

where *C* determines the lowest resolution of the velocity axis.

### 4.2 Denoising results

**Fig 7:**
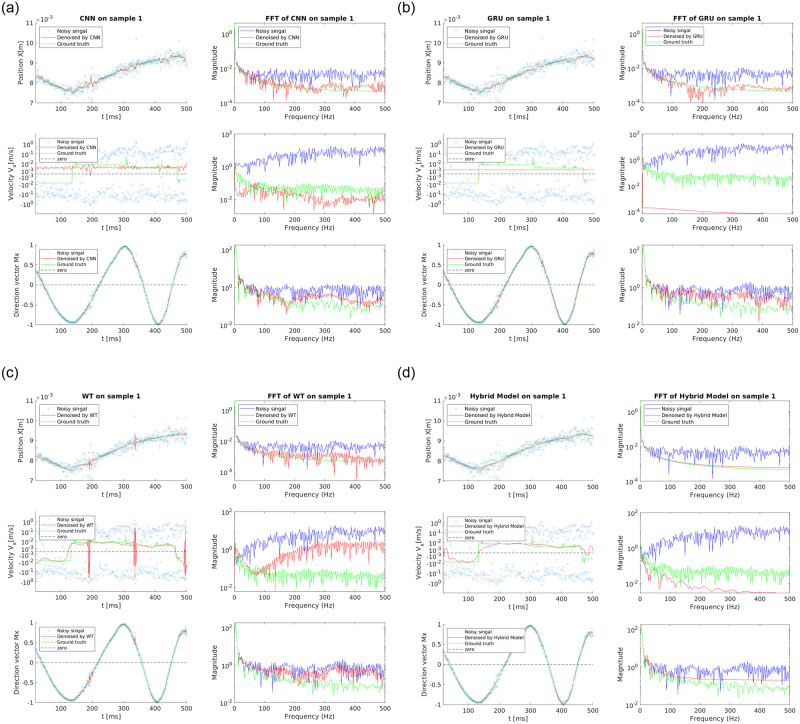
Comparison of the performance of a) CNN, b) GRU, c) WT, d) WT+GRU (hybrid model) on Sample 1 randomly drawn from the testing dataset.

**Fig 8:**
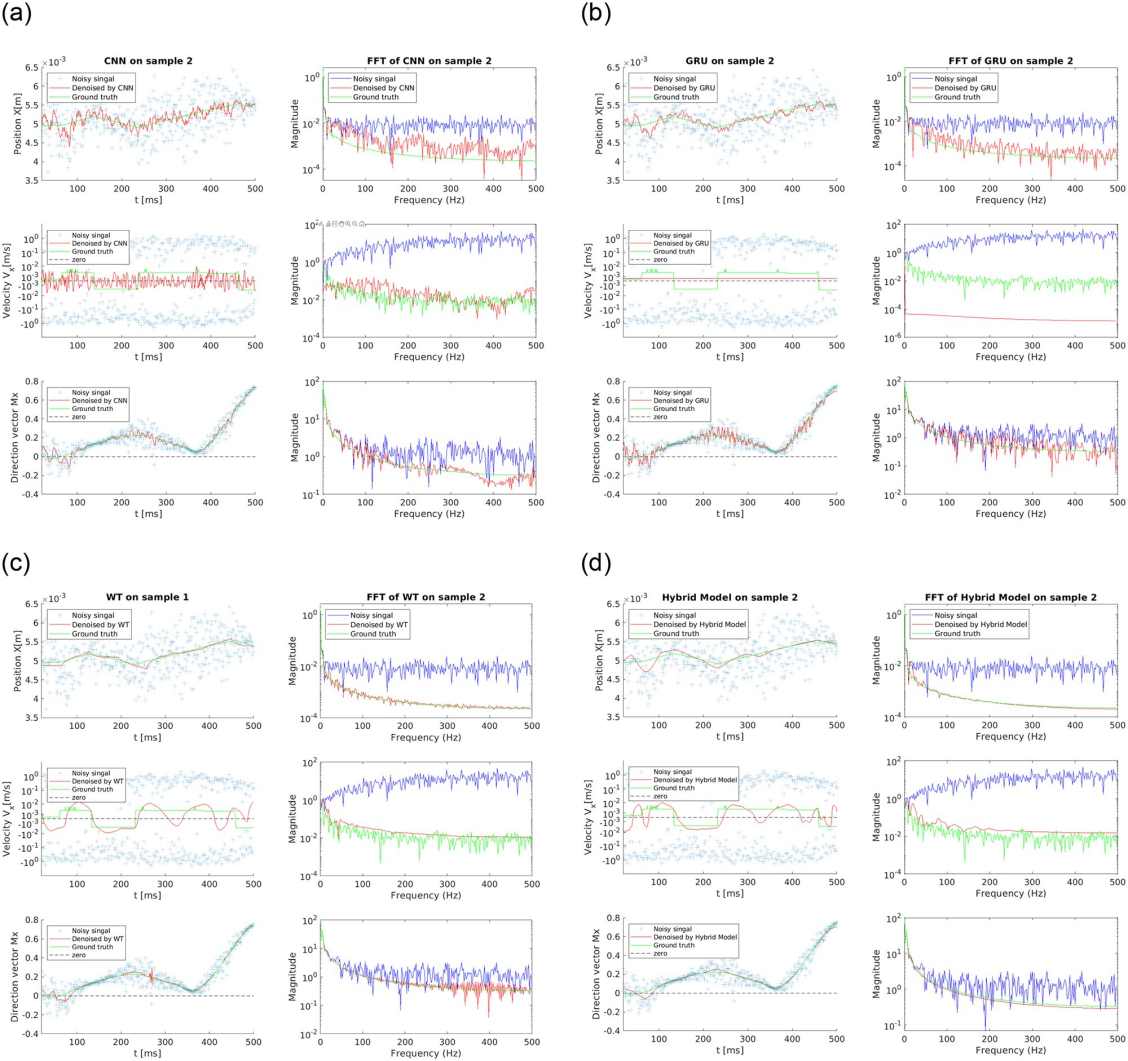
Comparison of the performance of a) CNN b) GRU c) WT d) WT+GRU (hybrid model) on Sample 2 randomly drawn from the testing dataset.

We utilize the above methods to denoise the position, velocity, and orientation of the magnetic particle. The denoiser performance is illustrated using two typical samples in the testing dataset (Figs 7 and 8). The ground truth is also plotted for comparison. The signals in both time and frequency domains are studied to evaluate the degrees of noise reduction and oversmoothing. For the position, all the proposed models provide results in good agreement with the ground truth. AI-based denoising methods display better performance than WT models when handling noise with large magnitude. However, large deviations from the ground truth still exist at some time intervals. The noise level and performance metrics are listed in Table 2. The CNN- and GRU-based algorithms outperform the WT model in terms of noise level, fluctuation level, as well as signal improvement ratio. Moreover, the GRU-based model suppresses both the noise and fluctuation better than the CNN-based model, which can be attributed to the inherent advantage of capturing long-term memory of sequential data. The Fast Fourier Transform (FFT) plots show that both the CNN and GRU-based models can significantly reduce the high-frequency noise of the trajectory signals; however, notable noisy components across the entire frequency domain still remain. In comparison, the WT method suppresses much less high-frequency noises. To further enhance the performance, a hybrid method is developed: first the WT filtering is used to preprocess the signal and reduce the noise, and then the GRU is applied on the filtered results to obtain the final velocity. By combining WT and GRU methods, the hybrid model yields the best denoised trajectory signal with a frequency distribution nearly identical to the ground truth. Hence the hybrid model stands out for denoising the position signal.

**Table 2:** Denoising models comparison.

Data	Noise measurement	Original	CNN	Improvement CNN	GRU	Improvement GRU	WT	Impro-vement WT	WT +GRU	Improvement WT+GRU
Position	Noise level	0.038	0.014	64.3%	0.013	65.3%	0.015	60.5%	0.010	74.5%
	Fluctuation level	36.32	4.924	86.4%	3.25	91.1%	6.758	81.4%	1.094	97.0%
Velocity	Noise level	36.322	0.79	97.8%	0.79	97.8%	5.904	83.7%	0.570	98.4%
	Fluctuation level	152.50	1.096	99.3%	1.001	99.3%	16.273	89.4%	1.043	99.3%
Orientation	Noise level	0.056	0.024	57.3%	0.043	22.4%	0.028	50.4%	0.025	54.7%
	Fluctuation level	4.37	0.70	83.9%	1.54	64.7%	1.514	65.3%	0.443	89.8%

The magnitude of velocity obtained from the noisy signal is 100 times larger than that of the clean signal. This poses a great challenge to velocity denoising, especially for the AI- based models because the magnitude of the true velocity signal is too small to be distinguished from numerical error. Surprisingly both AI-based models have lower noise and fluctuation levels as shown in Table 2. Figs 7 and 8 rectify our observation by showing that the AI-based models fail to capture the shape of the clean velocity signal in both samples. This indicates that the DNN model alone may not be able to reduce the noise without trimming off the velocity signal, which is a limitation of the neural network approach when directly applied on highly noisy data without any preprocessing (e.g., wavelet filtering). On the other hand, the WT method correctly captures the shape of the clean signal but fails to reduce large fluctuations in certain regions (Figs 7c and 8c). According to Figs 7d and 8d and Table 2, the hybrid model successfully attenuates the velocity fluctuations while retaining the shape of the signal. Note that the trajectories studied here are reconstructed using the 2-sensor setup. The burst of error issue as explained in Section 3.2 exists in these trajectories. Using more magnetic sensors can help completely remove the large fluctuation in certain regions and improve the accuracy. In addition, there still exist discrepancies between the shape of the denoised velocity and the clean signal in certain locations (e.g., the left end of velocity plot in Fig 7d), partially due to the fact that the velocity of synthetic trajectories contains Heaviside step functions. The discrepancies can be improved by modifying the preprocessing algorithm and increasing the depth of the DNN, which requires more training data and longer time. Moreover, it can be observed that the GRU-based model significantly oversmoothes the velocity signals, while the performance of the WT method is case-dependent. For sample 1, the WT model well recovers the frequency distribution at low frequency (<60 Hz), but it significantly deviates from the ground truth at higher frequencies (>60 Hz), indicating poor denoising performance. For sample 2, the WT-denoised result shows a good agreement with the ground truth. The hybrid model, though slightly oversmoothes the velocity signals, outperforms the method by GRU or WT model alone.

In terms of the orientation signal, all proposed models suppress the noise well, among which the CNN-based model has the best denoising performance. Interestingly, the denoising performance of the GRU-based model has been surpassed by the WT model. This is because the overall noise magnitude is small and large noise occasionally spikes up in random locations, which might confuse the GRU from a time sequence point of view. It is worth noting that the hybrid model has the best denoising performance for all variables (positions, velocity, and orientation) compared to AI-based models or the WT model, especially for velocity where a large noise magnitude is present.

## 5. Summary

This paper presents an analytical solution to the magnet particle positioning problem. Specifically, we can calculate the 3D position and orientation of a magnetic source based on the field vectors **B** at a few points, which are measured using 3-axis magnetometers. The solution allows us to develop a reconstruction algorithm for 3D particle tracking, which provides a novel approach to detect the flow in an opaque environment such as fluidized bed or turbidity flow. With zero noise, the reconstruction is exact. As the measurement noise increases, the mean reconstruction error grows linearly. For the two-magnetometer setup, the average position error is 3–4%, and orientation error is 5%, given that the measurement noise is 3%. The investigation of uncertainty propagation shows that at the same level of noise, the reconstruction error is highly non-homogeneous in the space and orientation domain, due to the high non-linearity of the magnetic dipole equation. The burst of error in certain regions can result in severe problems in denoising. Using more sensors can reduce the error.

In order to improve the reconstruction accuracy in practical scenarios where measurement noise is inevitable, we employ the CNN, GRU and WT-based methods to denoise the position, velocity, and orientation signals. All these methods lead to a significant improvement in the reconstruction accuracy. In the regions where the error is extremely large, the AI-based models provide better trajectory reconstruction. However, pure AI-based models fail to capture the shape of the velocity signals because the noise is 100 times larger than the true velocity magnitude. In contrast, the wavelet method can roughly capture the trend of the true velocity signal with a few exceptions. Therefore, we develop a hybrid approach that preprocesses the sequence by filtering out high-frequency noise with WT and then denoises the signal using GRU. The performance of the hybrid method outperforms the other denoisers in velocity denoising. Finally, all the methods perform well in the orientation denoising, and the CNN and hybrid models are slightly better. From the FFT analysis, we observe that the AI-based methods (GRU and CNN) tend to oversmooth the velocity signals, while the hybrid model can better capture the frequency distribution for most cases. In general, the hybrid method combining WT and GRU shows the best performance.

## List of symbols and abbreviations

### Symbol or name

ε: A small variable
σ: Eigenvalue of the error propagation matrix
β: The azimuthal angle between 2D solution and 3D field
γ: The noise variance in wavelet denoising
μ_0_: Magnetic permeability
|•|: The norm of a vector
**B**: Magnetic field vector
**C**: Scaling factor for discrete wavelet transform
g: Wavelet base function
h: Dipole field function (with a parameter)
*I*: Noise improvement ratio
i, j, k: Index, vector component, or integers
L: Distance between two magnetometers
**m** and *m*_*x*_ *m*_*y*_ *m*_*z*_: The magnet moment and its components
**M**: Normalized magnetic moment (two components)
**N** and **N**_**f**_: Noise level and noise fluctuation level
**n**: Unit vector along a certain direction
**P**: Error propagation matrix
**Q**: Number of signal elements in wavelet denoising
S, D, E and *f*: General variables or functions
**T**: Normalized
**V**: The state vector of a magnetic particle **V** = (*x y z m*_*x*_ *m*_*y*_ *m*_*z*_)
**W**: Invert of the error propagation matrix
***x*** and *x*, *y*, *z*: Position vector and its components
X, Y, Z: Coordinates in a 3D frame

### Abbreviation

CNN: Convolution neural network
DNN: Deep neural network
FFT: Fast Fourier transform
GRU: Gated recurrent unit
LSTM: Long short time memory
ReLU: Rectified linear unit
WT: Wavelet transform
